# Restless Legs Syndrome in Multiple Myeloma Patients

**DOI:** 10.4021/jocmr1083w

**Published:** 2012-09-12

**Authors:** Ramazan Esen, Levent Ediz, Elif Gulcu, Fahrettin Demirdag, Ugur Turktas, Savas Guner, Senar Ebinc, Adem Cifci, Cengiz Demir

**Affiliations:** a Yuzuncu Yil University Medical Faculty, Internal Medicine, Hematology Department, Van, Turkey; b Yuzuncu Yil University Medical Faculty, Physical Medicine and Rehabilitation Department, Van, Turkey; c Elazig Education and Research Hospital, Physical Medicine and Rehabilitation Department, Elazig, Turkey; d Yuzuncu Yil University, Medical Faculty, Orthopaedics and Traumatology department, Van, Turkey

**Keywords:** Restless legs syndrome, Multiple Myeloma, Depression, Anxiety, Frequency, Health related quality of life

## Abstract

**Background:**

Restless legs syndrome (RLS) is a common neurological condition characterized by uncomfortable and unpleasant sensations in the legs that are relieved by movement. It is frequently idiopathic, sometimes associated with specific disorders such as malignancies. Because there is no study relevant to RLS in Multiple Myeloma (MM), we aimed to evaluate the frequency of RLS in MM patients during chemotherapy and examined the relationship between presence of RLS and depression and anxiety in these patients.

**Methods:**

We enrolled a population of 62 adult MM patients for RLS features. RLS was ascertained in MM patients by both the presence of the four essential International RLS Study Group diagnostic criteria and neurological examination. The International RLS Study Group rating scale was used to measure RLS severity. Hospital Anxiety and Depression Scale (HADS) was used to evaluate the levels of depression and anxiety and Short Form-36 (SF-36) to evaluate health related quality of life (HRQOL).

**Results:**

A total of 62 MM patients were evaluated. Among them 11 were identified by the screening questionnaire to meet the criteria for RLS (17.74%). MM patients with RLS had higher levels of depression (P < 0.01) and anxiety (P < 0.01) and poorer HRQOL compared with those without RLS.

**Conclusions:**

The frequency of RLS in MM patients is higher than that of expected in the general population. MM patients afflicted by RLS have significantly higher levels of depression, anxiety and poorer HRQOL. Recognition and treatment of RLS in MM patients may be an important target in clinical management and may improve overall health outcomes in these patients.

## Introduction

Restless legs syndrome (RLS) is a sensory-motor disorder, characterized by paresthesias and intense urge to move lower extremities [1]. The disorder is idiopathic in 60% of the cases. It is considered secondary in 40% of the patients when linked to some diseases such as end-stage renal disease, pregnancy, rheumatic diseases, Parkinson’s disease, multiple sclerosis, and iron deficiency. RLS has a negative impact on sleep and quality of life. Dopamine dysfunction locally within the central nervous system (CNS) appears to get central role in the pathophysiology of the condition [1, 2]. The low brain iron is also a well recognized pathology of RLS [2].

Multiple myeloma (MM) is a neoplastic plasma B cell disorder that accounts for approximately 10% of all hematologic cancers [1, 2]. MM is characterized by overproduction of monoclonal immunoglobulins and the presence of this immunoglobulins in the serum and/or urine [3, 4]. It affects 4.3 in every 100,000 per year [5]. MM predominantly affects patients in the sixth and seventh decades [6]. The most common presenting symptoms are bone pain and fatigue. The primary cause of fatigue is anemia that occurs in 70% of MM patients at the diagnosis time. The hallmark of MM on radiologic skeletal survey is osteolytic bone lesions and/or compression fractures. Renal dysfunction is occurs in 50% of patients with MM and hypercalcemia in 25% [7]. Asymptomatic or symptomatic polyneuropathy may be observed in up to 50% of patients with plasma cell dyscrasias’s disease at the diagnosis time [8].

Up to date, the frequency of RLS in patients with MM has not been elucidated. Taking into account high prevalence of neuropathies, chemotherapeutic use and anemia in MM, we aimed to investigate the frequency of patients with MM under chemotherapy to suffer from RLS and examined the relationship between presence of RLS and quality of life, depression and anxiety in these patients.

## Materials and Methods

One hundred and twelve patients with MM under chemotherapy at the Department of Hematology, Yuzuncu Yil University, and age and sex matched healthy control subjects were included in this study. The study was approved by the medical ethical committee of Yuzuncu Yil University Medical Faculty, Van, Turkey and all patients gave written informed consent. A complete neurological examination was performed for all patients with MM; hypoesthesia, paresthesias, muscle weakness, abnormalities of deep tendon reflexes, and vibration threshold were evaluated for determining peripheral nervous system involvement. Data about demographic properties and medical history were recorded. The interview paper also covered the following questionnaire: The 4 essential criteria for RLS: 1) Irresistable urge to move the legs, usually associated with, or induced by, uncomfortable and unpleasant sensations in the lower limbs. 2) Irresistable urge to move or unpleasant sensations (symptom complex) begin or worsen during periods of rest or inactivity (when sitting or lying down). 3) Symptom complex is partially or totally relieved by movement, for instance when walking or stretching, at least as long as the activity continues. 4) Symptom complex is worse during the evening or night than during the day or only occurs in the evening or at night [9]. Those who meet all 4 criteria and if the frequency of experience of RLS symptoms was twice per week or more during the last 6 months were diagnosed as suffering from RLS. Then Epworth Sleepiness Scale (ESS) Turkish version was assessed to all RLS positive patients [10]. In the presence of RLS, RLS related symptom severity were detected using international RLS study group-rating scale (IRLSSG-RS) [11].

Levels of anxiety and depression were determined with Turkish version of Hospital Anxiety and Depression Scale (HADS), The HADS was translated into Turkish by Aydemir et al [12], satisfied validity and reliability studies and was reported as a suitable tool for the Turkish population. The reliability coefficient of the anxiety and depression HADS subscales for the Turkish patient group was 0.85 and 0.78, respectively [12]. HADS consists of two domains: one to assess anxiety status and the other to assess depression status. Short-Form 36-item survey (SF-36) was used to assess the health related quality of life (HRQOL). SF-36 has also been translated and validated in Turkish [13]. The HRQOL includes the following general health measures: physical functioning (PF), general health perceptions (GH), role limitations due to physical health problems (role-physical, RP), body pain (BP), fatigue or vitality (VT), social functioning (SF), role limitations due to emotional problems (role-emotional, RE), and mental health (MH). Higher scores represent better functioning.

### Statistical analysis

Statistical comparisons between MM patients and healthy subjects and between MM patients with RLS (MM(+)RLS) and MM patients without RLS (MM(-)RLS) were performed using Chi-square test or student t-test when appropriate. Results are reported as mean ± SD. Significance was set at P < 0.05.

## Results

Demographic properties of the MM patients and healthy controls were presented in Table 1. Sixty-two MM patients under chemotherapy (38 male and 24 female) included in the study. Mean age of MM patients was 56.6 ± 19.3 years (range 36 - 78). For other characteristics of the patients and healthy controls see Table 1. There was not any difference between MM patients and healthy controls with regard to age, gender and body mass index (BMI).

**Table 1:** Demographic Properties of the MM Patients and Healthy Control Subjects

	Patients included in the study (n = 62)	Healthy Controls (n = 62)
Age (year)	56.6 ± 19.3 (36 - 78)	56.3 ± 18.7 (35 - 76)
Gender	Male (n) = 38; Female (n) = 24	Male (n) = 37; Female (n) = 25
Body Mass Index (BMI)	23.4 ± 6.3	23.2 ± 6.5
ISS (International Staging System) stage	Stage I (n) = 6; Stage II (n) = 25; Stage III (n) = 28; Unknown (n) = 3	-
Myeloma type	IgG (n) = 34; IgA (n) = 18; Light chain (n) = 10	-
Presence of lytic lesion	n = 41	-

The frequency of RLS was 17.74% (11 out of 62) in patients with MM under chemotherapy. The frequency of RLS was significantly higher in patients with MM than in controls (P < 0.01). No significant differences were found between MM/RLS and Control/RLS patients with regard to ESS and IRLSSG-RS scores (P > 0.05) (Table 2).

**Table 2:** Restless Legs Syndrome (RLS) Frequency and IRLSSG-RS (International RLS Study Group-Rating Scale) and ESS (Epworth Sleepiness Scale) Scores in MM Patients and Healthy Control Subjects

	MM patients (n = 62)	Healthy Controls (n = 62)	P value
RLS diagnosis (n)	11 (17.74%)	2 (3.23%)	< 0.01^a^
ESS score	6.65 ± 4.39	6.69 ± 4.26	NS^b^
IRLSSG-RS Score	21.23 ± 6.43	20.98 ± 6.35	NS^b^

Data presented number (%) of patients or mean ± SD. ^a^Chi-Square test; ^b^Student’s t-test and Mann-Whitney U-test; NS, not statistically significant (P > 0.05).

Significant differences were found between MM/RLS and MM/non-RLS patients with regard to HADS-Anxiety and Depression scores. The occurrence of RLS in MM patients was associated to significantly higher levels of depression (P < 0.01) and anxiety (P < 0.01) (Table 3). The relationship between the presence of RLS in MM patients and HRQOL is also shown in Table 3. RLS-affected MM patients reported lower physical functioning (P < 0.01), lower emotional well-being (P < 0.01) and lower mental well-being (P < 0.01) than unaffected patients.

**Table 3:** Comparison of HADS (Hospital Anxiety and Depression Scale) Scores and SF-36 Subscales Between MM Patients With and Without RLS

	MM (RLS +)	MM (RLS -)	P value
HADS Depression	9.1 ± 4.5	6.7 ± 4.0	< 0.01
HADS Anxiety	9.2 ± 4.2	6.6 ± 4.2	< 0.01
SF-36 Physical Functioning (PF)	42.07 ± 11.56	44.56 ± 11.34	< 0.01
SF-36 Role-Physical (RP)	45.02 ± 7.96	45.82 ± 10.57	NS
SF-36 Body Pain (BP)	49.78 ± 8.71	49.22 ± 8.96	NS
SF-36 General Health (GH)	48.28 ± 8.19	48.42 ± 9.32	NS
SF-36 Vitality (VT)	55.23 ± 9.57	55.43 ± 9.75	NS
SF-36 Social Functioning (SF)	44.05 ± 10.14	44.02 ± 9.98	NS
SF-36 Role-Emotional (RE)	41.83 ± 11.65	44.69 ± 11.34	< 0.01
SF-36 Mental Health (MH)	45.59 ± 10.77	48.28 ± 10.89	< 0.01

## Discussion

This study shows that a very high proportion of MM patients under chemotherapy (17.74%) concurrently suffer from RLS. MM patients afflicted by RLS also have significantly higher levels of depression, anxiety and poorer HRQOL. To our knowledge, this is the first study showing the increased frequency of RLS in patients with MM. The prevalence of RLS in the general population has been said to be between 3% and 9% [1, 2]. In the current study, we found that 3.23% of the healthy Turkish population had RLS.

It is not clear how to explain a possible relationship between MM and RLS. The low brain iron may be a reason. A majority of MM patients has anemia. Iron is a necessary cofactor for the synthesis of dopamine. Dopamine dysfunction locally within CNS gets central role in the pathophysiology of RLS. In the current study, we did not find any difference between MM/RLS patients and MM/non-RLS patients in terms of iron and ferritin deficiency. Another possible relationship could be neurochemical predisposition due to chronic pain nature of MM. Because 64% of patients diagnosed with Fibromyalgia Syndrome which is a rheumatologic and chronic pain condition, also concurrently suffered from RLS [14].

These observations may suggest a possible link between chronic pain and RLS predisposition in MM patients. Another possible relationship between MM and RLS may be due to polyneuropathy seen in MM. RLS is frequent in acquired polyneuropathy of sensory type and mild entity. RLS was present in 30% of patients with acquired polyneuropathy [15]. Electrophysiologically diagnosed neuropathy is common in MM patients under chemotherapy [8]. Polyneuropathy may also be a reason of increased RLS frequency in MM.

In conclusion, the frequency of RLS in MM patients is higher than that of expected in the general population. MM patients afflicted by RLS have significantly higher levels of depression, anxiety and poorer HRQOL. Recognition and treatment of RLS in MM patients may be an important target in clinical management and may improve overall health outcomes in these patients. Further studies are needed to clarify the features and pathogenetic mechanisms underlying MM-related RLS.
